# Overexpression of bicarbonate transporters in the marine cyanobacterium *Synechococcus* sp. PCC 7002 increases growth rate and glycogen accumulation

**DOI:** 10.1186/s13068-020-1656-8

**Published:** 2020-01-28

**Authors:** Jai Kumar Gupta, Preeti Rai, Kavish Kumar Jain, Shireesh Srivastava

**Affiliations:** 1 Systems Biology for Biofuels Group, International Centre for Genetic Engineering and Biotechnology (ICGEB), ICGEB Campus, Aruna Asaf Ali Marg, New Delhi, 110067 India; 2 DBT-ICGEB Centre for Advanced Bioenergy Research, New Delhi, India

**Keywords:** Feedstock, Photosynthesis, Marine, Carbohydrate, Biomass

## Abstract

**Background:**

*Synechococcus* sp. PCC 7002 is an attractive organism as a feedstock and for photoautotrophic production of biofuels and biochemicals due to its fast growth and ability to grow in marine/brackish medium. Previous studies suggest that the growth of this organism is limited by the HCO_3_^−^ transport across the cytoplasmic membrane. Tools for genetic engineering are well established for this cyanobacterium, which makes it possible to overexpress genes of interest.

**Results:**

In this work, we overexpressed two different native Na^+^-dependent carbon transporters viz., SbtA and BicA in *Synechococcus* sp. PCC 7002 cells under the influence of a strong light-inducible promoter and a strong RBS sequence. The overexpression of these transporters enhanced biomass by about 50%, increased intracellular glycogen about 50%, and increased extracellular carbohydrate up to threefold. Importantly, the biomass and glycogen productivity of the transformants with air bubbling was even higher than that of WT cells with 1% CO_2_ bubbling. The overexpression of these transporters was associated with an increased carotenoid content without altering the chl *a* content.

**Conclusions:**

Our work shows the utility of increased carbon transport in improving the growth as well as product formation in a marine cyanobacterium and will serve to increase the utility of this organism as a potential cell factory.

## Background

Cyanobacteria are attractive organisms for the production of biofuels, biomass and other bioproducts due to their ability to carry out photosynthesis as well as their genetic tractability [1]. They also do not compete for land, like terrestrial plants. Marine strains are useful as they do not use fresh water, which is getting scarce in many parts of the world. However, the use of these microorganisms as a potential cell factory can be significantly boosted by increasing their photoautotrophic growth [2]. Previous studies have suggested that the transport of carbon is the major limiting factor for the growth of cyanobacteria. Various strategies such as addition of sodium bicarbonate, medium optimization, and genetic engineering of genes such as carbonic anhydrase, etc., have been shown to increase the growth [3–5].

*Synechococcus* sp. PCC 7002 is one of the fastest-growing marine cyanobacteria, with reported photoautotrophic doubling times of ~ 2.6 h [6]. Earlier studies have shown that bubbling cyanobacterial cultures with 1% to 3% CO_2_ in air results in improved growth rates [7, 8]. This indicates that the growth rate is limited by the carbon availability and transport across the cytoplasmic membrane. There are three major types of bicarbonate transporters in cyanobacterial cells, which differ in their affinity to HCO_3_^−^/CO_2_: (a) BCT1, an ATP-binding cassette (ABC) transporter, a medium affinity low flux transporter that was the first reported bicarbonate transporter [9], (b) the sodium-dependent bicarbonate transporter A, an inducible, high affinity, low flux transporter Sbt A, and (c) the bicarbonate transporter Bic A, a constitutive, low affinity, high flux transporter. There is an upregulation of transcripts of *bicA* and *sbtA* in *Synechococcus* sp. PCC 7002 under C_i_-limiting conditions, indicating that these genes play a major role in C_i_ uptake [10]. Both SbtA and BicA are sodium-dependent active bicarbonate transporters that require about 1 mM Na^+^ for their half-maximal HCO_3_^−^ transport activity [11, 12]. SbtA is a homo-tetramer of approximately 160 kDa [13], while BicA is a monomeric transporter of 60 kDa [14]. Previously, BicA-overexpression in the freshwater cyanobacterium *Synechocystis* sp. PCC 6803 under the control of the nirP promoter was shown to improve growth [15]. The knock-out of both *sbtA* and *bicA* genes showed significantly reduced bicarbonate transport and slower growth at pH 9.3 [12].

A major advantage of cyanobacteria is the ease of genetic engineering, at least in some well-studied strains such as the *Synechococcus* sp. PCC 7002, PCC 7942 and *Synechocystis* sp. PCC 6803 cells. The availability of synthetic biology toolbox for *Synechococcus* sp. PCC 7002 [16], makes it possible to genetically engineer this strain and regulate the expression of the target gene(s). Some examples of successful genetic engineering of cyanobacteria include genetic engineering for heparosan [17] and isobutanol production [18], in addition to direct photosynthetic production of biofuels and bioproducts [19]. However, *Synechococcus* sp. PCC 7002 has very few promoters of known strength and only one reported inducible promoter [20, 21]. Previous studies have compared the strengths of variants of a strong light-inducible promoter of the large subunit of Rubisco, P_rbcL2A_ from *Synechocystis* sp. PCC 6803 cells [22], as well as various ribosome binding sites (RBS) in *Synechococcus* sp. PCC 7002 cells [16].

In the present study, we demonstrate generation of two transgenic strains of *Synechococcus* sp. PCC 7002 overexpressing native *sbtA* and *bicA* genes under the control of the promoter and RBS identified in the previous studies [16, 22]. Our work shows significant improvements in cell growth and glycogen content of the cells upon overexpression of bicarbonate transporters.

## Results

### Evaluation of the transformation

**Fig. 1:**
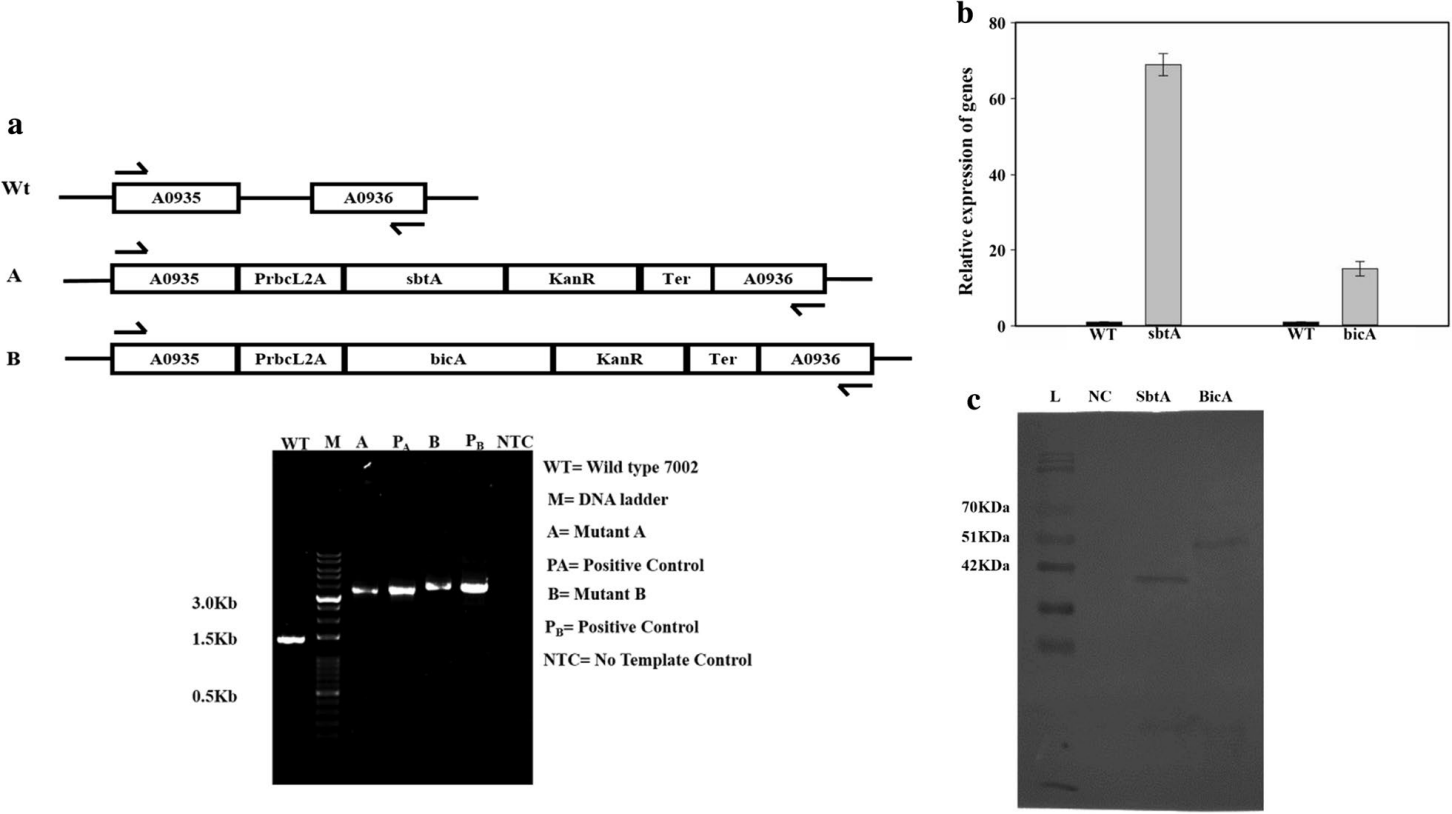
Evaluation of transformation through PCR. **a** The *sbtA* and *bicA* gene constructs, having promoter and antibiotic selection marker were incorporated between NSI and NSII by double homologous recombination. Arrows indicate the primers used for PCR amplification of the region between NSI and NSII sites. WT, genomic DNA from the wild-type cells was used as the template; M, molecular weight marker; A, genomic DNA from strain A (*sbtA*-overexpressing strain) was used as the template; pA, plasmid pA was used as the template; B, genomic DNA from strain B (*bicA*-overexpressing strain) was used as the template; pB, plasmid pB was used as the template; NTC, no-template control, only buffer used as the template. **b** Measurement of relative levels of *sbtA* and *bicA* mRNA using qRT-PCR. *N* = 3. **c** Measurement of the expression of SbtA and BicA proteins using Western blotting using an anti-His6 antibody. L, Protein marker; NC, negative control, proteins extracted from the wild-type cells; A, proteins extracted from strain A; B, proteins extracted from strain B

The successful transformation was evaluated through the amplification of the insert from the genomic DNA of the WT and transformed cells, as shown in Fig. 1a. The transformed cells showed a larger PCR product, demonstrating the integration of the insert at the desired site (Fig. 1a). Among the transformants, B showed larger inserts due to the larger size of the *bicA* gene. A list of strains and plasmids generated is given in Table 1.

**Table 1:** List of strains and plasmids used in the study

	Description
***Strain***	
Wt 7002	Wild-type *Synechococcus* sp. PCC 7002
A	Mutant 7002 having SbtA transporter
B	Mutant 7002 having BicA transporter
***Plasmid***	
pSK+	pBlueScript II SK(+) as backbone vector
pA	Plasmid having *sbtA* gene cassette
pB	Plasmid having *bicA* gene cassette

Five colonies of PCR-analyzed transformants were inoculated in liquid A^+^ medium and the growth rates were found similar among the colonies of individual transformants (data not shown).

Transformants A and B were also analyzed at RNA and protein levels, relative to wild type. mRNAs of *sbtA* and *bicA* were highly upregulated in the transformants A and B, respectively. Relative expression of *sbtA* was found 67 ± 3-folds higher in the transformant A (Fig. 1b) and was 15 ± 1.9-fold higher for the *bicA* gene in the transformant B (Fig. 1b). Western blot results indicate that the SbtA and BicA proteins are successfully expressed. Bands for the SbtA and BicA were observed at ~ 35 kDa and ~ 50 kDa, respectively (Fig. 1c). However, the bands do not correspond exactly to the molecular weights of the proteins, as is often observed with membrane proteins [15].

### Growth of WT and transformed cells at different light intensities and different CO_2_ concentrations

**Fig. 2:**
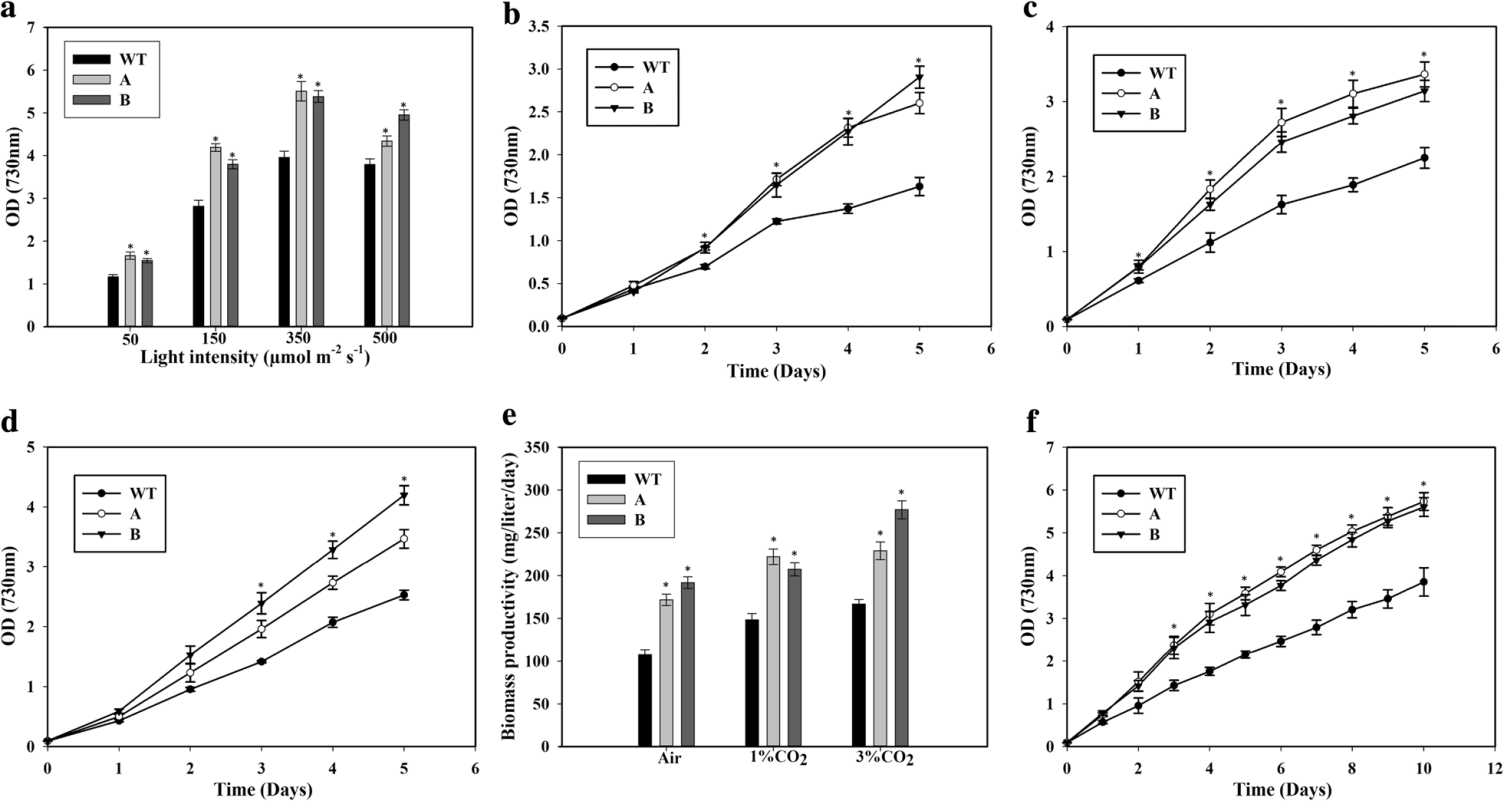
Growth of wild type (WT), SbtA-overexpressing (A) and BicA-overexpressing (B) strains. **a** The response of the cells to different light intensities. This experiment was done in a multi-cultivator (MC1000-OD) with air bubbling. The OD after 5 days of growth at different light intensities was measured. **b** Growth curve in Dreschel (gas-washing) bottles with air bubbling. *p < 0.05 for both the strains A and B compared to WT cells for the same day. **c** Growth curve with 1% CO_2_ bubbling. *p < 0.05 for both the strains A and B compared to WT cells for the same day. **d** Growth curve with 3% CO_2_ bubbling. *p < 0.05 for both the strains A and B compared to WT cells for the same day. **e** Biomass productivity with the bubbling of air, 1% or 3% CO_2_ after 5 days of growth. *p < 0.05 compared to WT cells for the same CO_2_ level. **f** Growth of cells in long-term culture with 1% CO_2_ bubbling. *p < 0.05 for both the strains A and B compared to WT cells for the same day. *n* = 2 for the long-term culture, *n* = 3 for all the other experiments

For studying the response of the cells to different light intensities, the cells were grown in a multi-cultivator (MC-1000 OD, PSI instruments, Czech Republic). As shown in Fig. 2a, the growth of the cells increased with the light intensity until 350 μmol m^−2^ s^−1^. However, a further increase in the light intensity did not promote the growth of the WT cells, while the increase was detrimental to the growth of the transformed cells (Fig. 2a). This intensity of 350 μmol m^−2^ s^−1^ is within range of the intensity previously used for WT *Synechococcus* sp. PCC 7002 cells [8]. The engineered cells showed significantly better growth compared to the WT cells at all light intensities tested. An intensity of 350 μmol m^−2^ s^−1^ was used for further studies.

Further experiments with higher CO_2_ concentrations were conducted in Dreschel (gas-washing) bottles equipped with gas dispersion tubes (Sigma Aldrich Chemical Co.) and kept in a water bath with light coming from the top. The transformed cells overexpressing SbtA and BicA transporters exhibited significantly improved growth as compared to the WT cells when the cultures were bubbled either with air, or 1% CO_2_ (Fig. 2b, c). When cultured with air bubbling, the transformant A showed about 60% higher OD than WT, while the transformant B showed 75% higher OD (Fig. 2b). With 1% CO_2_ bubbling, both the transformants showed about 50% higher OD compared to WT cells (Fig. 2c). Importantly, the OD of the transformants with air bubbling was even higher than that of WT cells with 1% CO_2_ bubbling. There was no significant difference in OD between the transformants with either 1% CO_2_ or air bubbling. When cultured with an elevated CO_2_ concentration of 3% too, the transformants showed higher growth compared to the WT cells (Fig. 2d). The WT cells and the transformant A showed a non-significant difference in growth at 3% CO_2_ compared to 1% CO_2_. However, the transformant B showed a significant increase in biomass accumulation when cultured with 3% CO_2_ compared to the culture at 1% CO_2_. The growth of transformant B was more than that of the transformant A at 3% CO_2_.

The cell dry cell weight per OD was about the same for all the three strains at 0.33 ± 0.03 g L^−1^ OD^−1^. This value was used to calculate the biomass productivity of the three strains. Biomass productivity of the WT cells increased with higher CO_2_ concentration, with values of 107.6 ± 5.7, 148.2 ± 7.1 and 166.8 ± 5.4 mg L^−1^ day^−1^ on air, 1% CO_2_, and 3% CO_2_, respectively (Fig. 2d). Thus, while there was a strong increase from air to 1% CO_2_, the increase from 1% CO_2_ to 3% CO_2_ was less drastic. The transformants showed greater biomass productivity compared to the WT at all the CO_2_ concentrations tested. Strain A (*sbtA* transformant) had a productivity of 171.7 ± 6.6 mg L^−1^ day^−1^, 222 ± 9 mg L^−1^ day^−1^ and 229 ± 10.2 mg L^−1^ day^−1^ in air, 1% CO_2_ and 3% CO_2_, respectively. Thus, strain A showed a significant increase in productivity from air to 1% CO_2_, but no further increase when cultured in 3% CO_2_. Strain B had the highest productivity in the air of about 191.8 ± 6.9 mg L^−1^ day^−1^, which increased only slightly in 1% CO_2_ (207.3 ± 7.6 mg L^−1^ day^−1^), but increased significantly in 3% CO_2_ (277 ± 10.5 mg L^−1^ day^−1^). Importantly, the biomass productivity of the transformants in the air was comparable or better than that of the WT cells even in 3% CO_2_.

Longer cultures up to 10 days on air showed a similar trend. The biomass accumulation of the transformants was about 60–70% higher throughout (Fig. 2e).

### Glycogen content and productivity

**Fig. 3:**
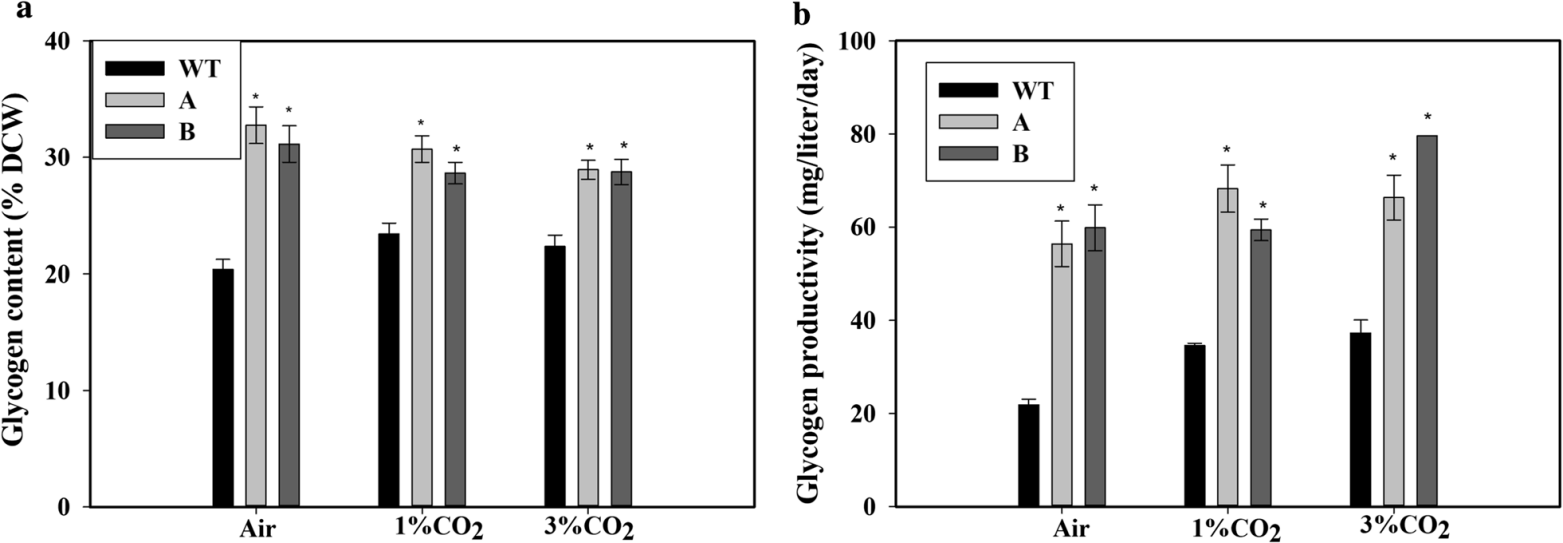
Glycogen content and productivity of the cells. **a** Intracellular glycogen content, **b** glycogen productivity of wild type (WT), SbtA-overexpressing (A) and BicA-overexpressing (B) cells with bubbling of air, 1% CO_2_ or 3% CO_2_, measured after 5 days of growth. Graph bars with * represent statistically significant difference (*p* < 0.05) with wild-type cells grown at the same CO_2_ concentration, *n* = 3

Because glycogen is a major storage molecule of the cyanobacterial cells, we also measured the glycogen content of the cells. After 5 days of growth on air bubbling, the glycogen content was 33% ± 1.9% (of DCW) in the transformed strains and only 20% ± 1% (of DCW) in WT with air bubbling (Fig. 3a). A similar response of increased glycogen content in the strains A and B compared to the WT cells was observed when the cells were cultured with 1% CO_2_ or 3% CO_2_ bubbling (Fig. 3a). Unlike the increase in growth at higher CO_2_ concentration, there was no significant change in glycogen content of the cells with increasing CO_2_ concentration. Therefore, the glycogen productivity with air bubbling was increased ~ 175% in strain B compared to WT (Fig. 3b). Even at higher CO_2_ concentrations of 1% and 3%, the glycogen productivity of the transformants was about double that of the WT cells.

### Pigment content of the transformed cells

**Fig. 4:**
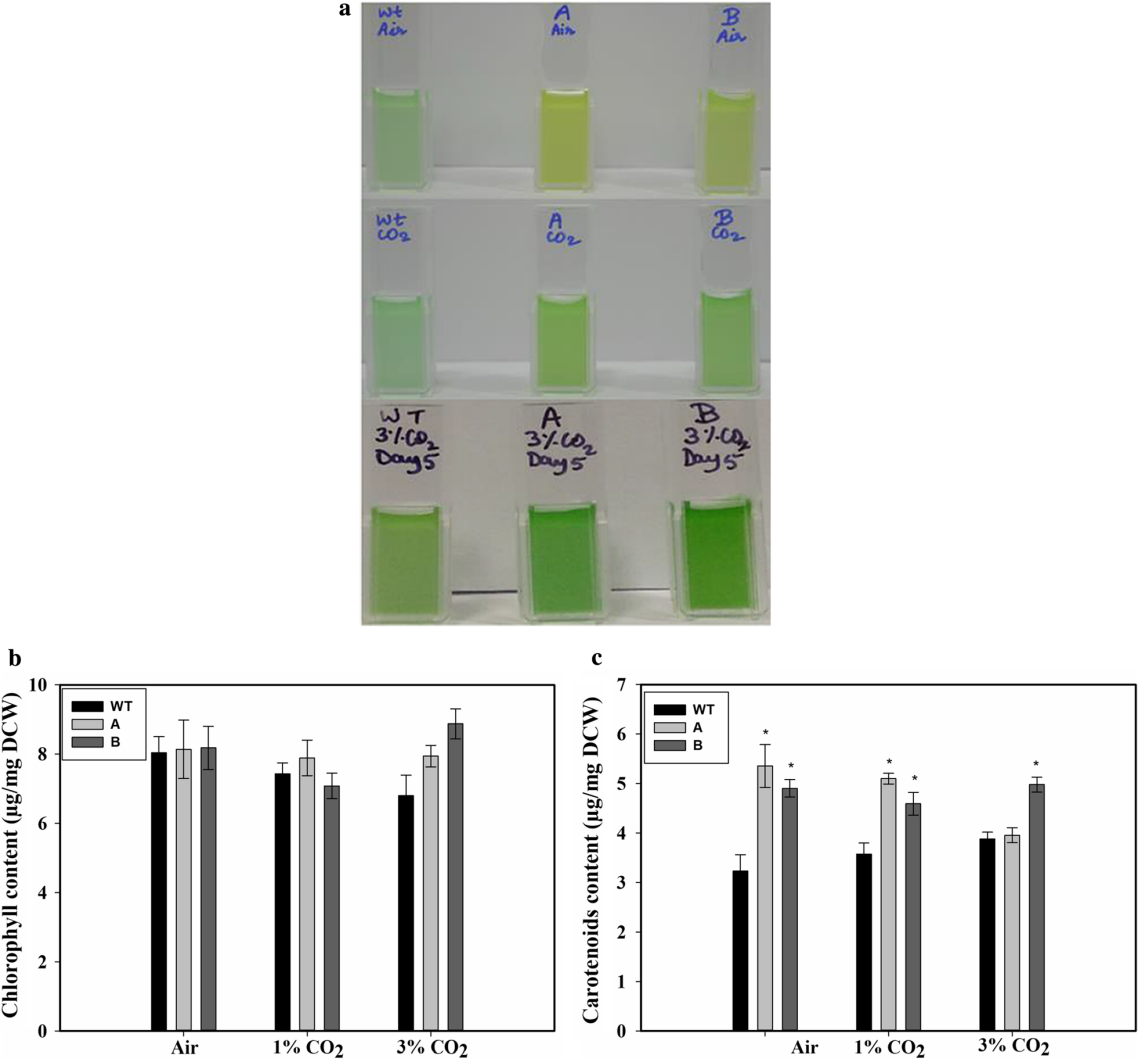
Appearance and the content of pigments in the wild-type and the transformed strains. **a** Appearance of the WT, SbtA-overexpressing (A) and BicA-overexpressing (B) cells when grown with air bubbling (upper panel), bubbling of 1% CO_2_ (middle panel) and bubbling of 3% CO_2_ (bottom panel). **b** Chlorophyll content, and **c** carotenoids content, after 5 days of growth with the bubbling of air, 1% CO_2_ or 3% CO_2_. * represents statistically significant difference from the wild-type cells grown under similar CO_2_ level, *n* = 3

The transformed cells appeared paler than the WT cells when grown under air bubbling (Fig. 4a, top panel). However, no significant difference in appearance was observed when the cells were grown in the presence of 1% or 3% CO_2_ (Fig. 4a, middle and bottom panels). Measurement of the chlorophyll *a* indicated that there was no significant difference in chl *a* content among the WT and transformants, when cultured either with the bubbling of air or of 1% CO_2_ (Fig. 4b). However, strain B showed an elevated level of chl *a* when grown in the presence of 3% CO_2_ (Fig. 4b). There was a significant increase of up to ~ 60% in the content of carotenoids (accessory photosynthetic pigments) in the transformed cells when grown in the presence of air (Fig. 4c). Increased carotenoids were observed in both the transformed strains at 1% CO_2_ as well. However, when grown at a 3% CO_2_ level, only strain B showed elevated carotenoid content.

### Effect on phycobiliproteins levels

**Fig. 5:**
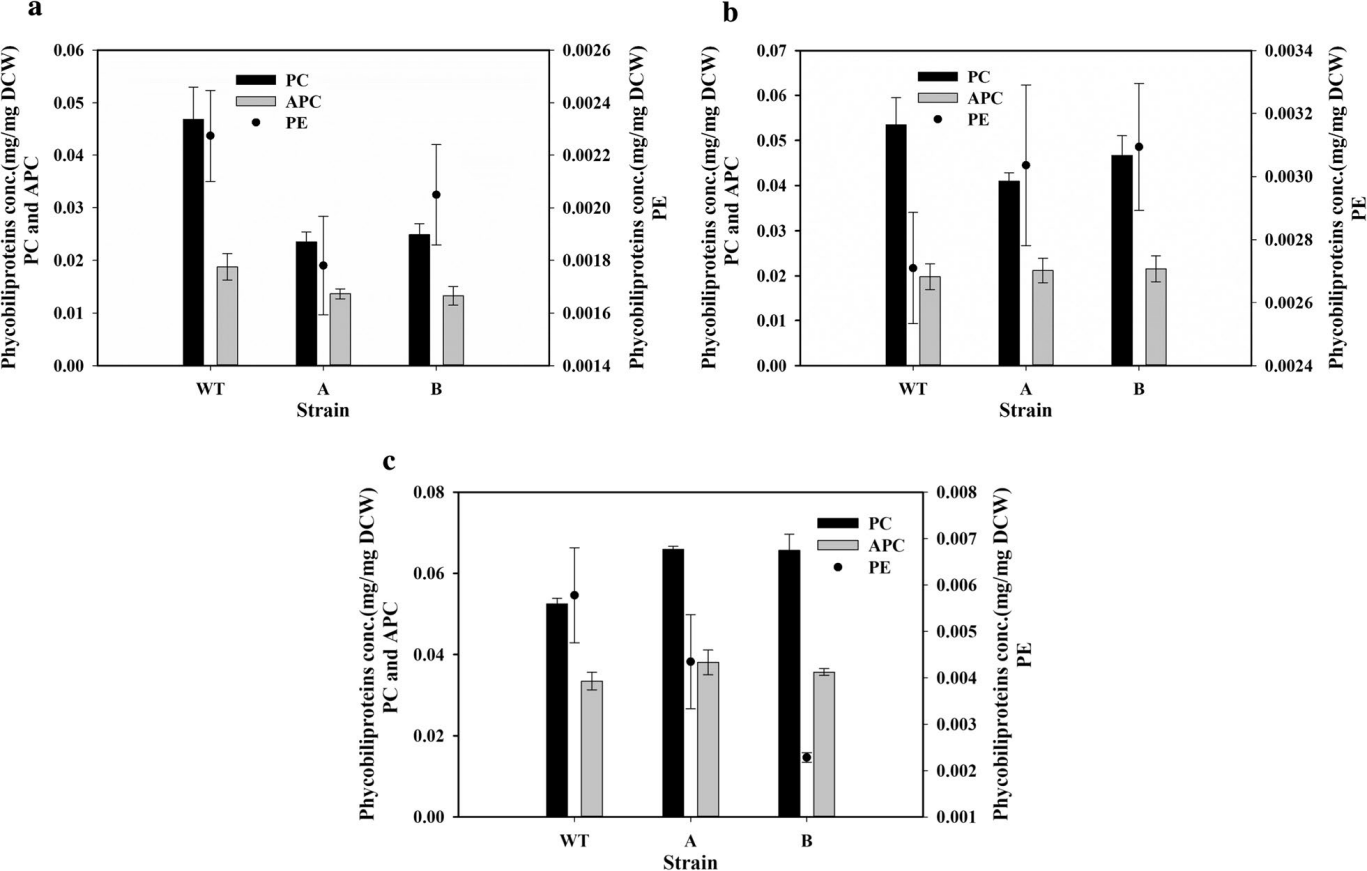
Phycobiliproteins content of the cells. The phycobiliprotein content of the wild type (WT), SbtA-overexpressing (A) and BicA-overexpressing (B) cells was measured after 5 days of growth with **a** air bubbling, **b** 1% CO_2_ bubbling and **c** 3% CO_2_ bubbling. *n* = 3 for all the figures

Phycobiliproteins (PBPs) were found to be higher in wild-type strain when compared to transformants grown on air. Phycocyanin (PC), the most abundant phycobiliprotein, was reduced to about half in the transformed cells with air bubbling (Fig. 5a). Reductions were also observed in the lesser-abundant allophycocyanin (APC) and phycoerythrin (PE) (Fig. 5a). When the cells were grown with 1% CO_2_ bubbling, the reduction in PBPs was much less, with only PC showing a slight reduction (Fig. 5b). There was an increase in the PC content of the transformed cells, while APC showed no difference and PE showed a reduction when the cells were grown in 3% CO_2_ (Fig. 5c).

### Effect on extracellular carbohydrate

**Fig. 6:**
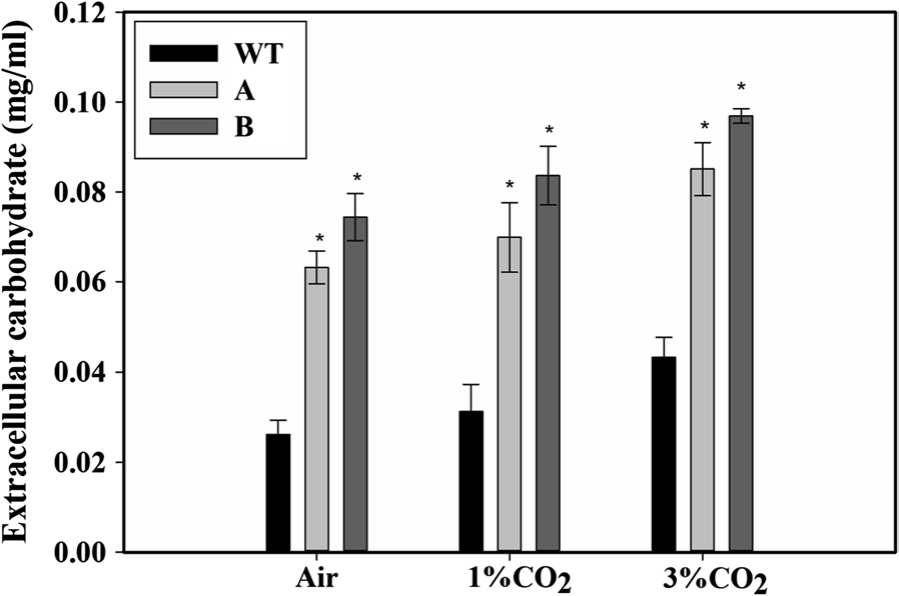
Measurement of the extracellular carbohydrate in the culture medium. Extracellular carbohydrate measured in the culture medium of the wild type (WT), SbtA-overexpressing (A) and BicA-overexpressing (B) strains was measured after 5 days of growth with bubbling of air, 1% CO_2_ or 3% CO_2_. * represents a statistically significant difference from the wild-type cells grown under similar CO_2_ level, *n* = 3

It was observed that there was a slight increase in the extracellular carbohydrate with increasing CO_2_ concentration (Fig. 6). In all the CO_2_ concentrations tested, strain A had double or higher extracellular carbohydrate levels compared to the WT cells, while strain B had even higher extracellular carbohydrate levels (Fig. 6).

## Discussion

The light intensity chosen for the culture of cyanobacterial cells in a bioreactor is optimized to provide a balance between self-shading and photobleaching. *Synechococcus* sp. PCC 7002 cells are relatively resistant to high light intensity and even an intensity of up to 600 μmol m^−2^ s^−1^ was shown to not significantly affect growth. We have used an intensity of 350 μmol m^−2^ s^−1^ which was found to be optimal for the WT and transformant cells. A number of studies in cyanobacteria and algae have shown enhanced growth and carbohydrate content of the cells upon addition of bicarbonate to the medium [23–26] which can be another strategy to increase biomass production.

We have successfully over-expressed functional sodium-dependent bicarbonate transporters SbtA and BicA in the marine cyanobacterium *Synechococcus* sp. PCC 7002 for the first time. We have used a light-dependent promoter P_rbcL2A_ in the marine cyanobacterium *Synechococcus* sp. PCC 7002. This promoter has been shown to have a higher activity than other commonly used promoters tested [20]. The biomass production of the transformed cells was increased significantly compared to the WT cells in both medium-term (5 days) and long-term cultures (10 days). Growth in the presence of elevated concentrations of CO_2_ increased the biomass accumulation in both WT and the transformed cells. The growth of both the transformants with air-bubbling was even better than the growth of WT cells under 1% CO_2_. Thus, the transformants showed enhanced biomass production without the need to increase CO_2_ concentration. The transformants also showed increased biomass productivity at higher CO_2_ concentrations, indicating better performance at all the CO_2_ concentrations tested. The improvement in the growth of the BicA transformants in our study is comparable with that reported earlier for the freshwater strain *Synechocystis* sp. PCC 6803 [15]. However, the effect on glycogen accumulation was not reported in the earlier study [15].

The biomass accumulation was almost similar among the two transformants under the various conditions tested, suggesting that carbon transport is similar between the two transformants. However, there were some differences in the response of the two transformants. For example, at 3% CO_2_ concentration the growth of the *bicA*-transformant was better than the *sbtA*-transformant (Fig. 2d), which also had a higher content of chl *a* and carotenoids at this CO_2_ concentration (Fig. 4b, c). However, in spite of the similar levels of chlorophyll content among the cells, the growth rate of the transformed cells was higher than those of the WT cells.

**Fig. 7:**
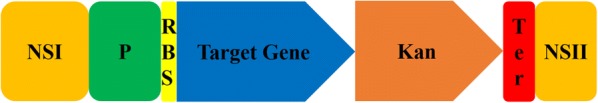
A pictorial representation of the generalized cassette used for genetic engineering. The neutral site I (NSI), promoter (P), RBS, bicarbonate transporter gene (target gene), kanamycin resistance gene (Kan), terminator (Ter) and neutral site II (NSII)

Cyanobacteria store glycogen, while other photosynthetic organisms store starch or β-glucans as the intracellular carbon-storage polymer [27]. Because of their smaller particle size (0.04–0.05 μm) and higher water solubility than the starch particles (0.1–100 μm) [27, 28], glycogen can be a better feedstock source for fermentation [27]. Also, due to greater branching in glycogen compared to starch, its conversion to simple sugars is much easier due to a greater surface area for hydrolysis [28]. One of the important findings of this study is that the transformed cells accumulated high amounts of glycogen compared to the WT cells (Fig. 4). Glycogen accumulation (% dry cell weight) was found to be increased by up to 50% while glycogen productivity (milligrams per liter per day) was increased up to 175%, respectively, in the transformed cells relative to WT cells under the same conditions. Additionally, the transformants showed up to a threefold increased accumulation of extracellular carbohydrates compared to the WT (Fig. 7). The increased extracellular carbohydrate content is in agreement with the previous study on BicA-overexpression in *Synechocystis* sp. PCC 6803 cells supplemented with 10 mM and 100 mM HCO_3_^−^ [15]. However, transformants A and B generated in our study showed a higher extracellular carbohydrate content (Fig. 6). The presence of high levels of extracellular carbohydrate indicates a greater metabolic overflow under the C-surplus conditions [5, 15]. Interestingly, when either the WT or the transformants were grown on higher CO_2_ concentration, there was no increase in glycogen content compared to when grown on air (Fig. 3a) in spite of an increase in growth (Fig. 2c). This shows a divergent metabolic response to HCO_3_^−^ vs. CO_2_. It is expected that the increased Ci intake is also associated with altered intracellular metabolite pools such as increased cellular ATP and NADPH levels which play a role in the increased growth rates observed in the transformants. The measurement of such metabolic changes will be the focus of subsequent studies.

Transformed cells showed significantly higher content of carotenoids when grown on air or 1% CO_2_. The level of phycocyanin (the most abundant phycobiliprotein) did not vary with CO_2_ concentration in the WT cells, but increased with the CO_2_ concentration in the transformed cells. These results indicate a complex interrelationship between the carbon transport and the content of the pigments in the *Synechococcus* PCC 7002 cells.

## Conclusions

Our results show significant improvements in growth and glycogen content of the cells overexpressing the bicarbonate transporters. The growth profiles of the transformants were generally comparable. The improved growth and glycogen productivity is an important development in the direction of utilizing marine cyanobacterium for biotechnological and feedstock purposes.

## Materials and methods

### Materials

The chemicals used in this study were purchased from Fisher Scientific (NaCl, Tris base), Amresco (KCl, ampicillin disodium salt, and kanamycin monosulfate, bacteriological agar), and Sigma-Aldrich (MgSO_4_·7H_2_O, CuSO_4_·5H_2_O, Na_2_EDTA, H_3_BO_3_, CaCl_2_·2H_2_O, KH_2_PO_4_, NaNO_3_, vitamin B_12_, FeCl_3_·6H_2_O, MnCl_2_·4H_2_O, ZnCl_2_, CoCl_2_·6H_2_O, RNaseZAP). Primers were synthesized by Sigma-Aldrich. GenElute Bacterial Genomic DNA kit from Sigma-Aldrich was used to isolate cyanobacterial genomic DNA. Other kits utilized in the molecular biological methods were GeneJET Plasmid Miniprep, GeneJET PCR purification, and GeneJET Gel Extraction DNA Recovery kits, FD restriction enzymes, DNA polymerases, and ligases, all purchased from Thermo-Scientific. Suppliers of all other materials used in this study are described below.

### Cyanobacterial strains and culture conditions

The wild-type *Synechococcus* sp. PCC 7002 used in this study was obtained from the Pasteur culture collection (PCC), Paris, France. Primary cultures were grown in 250-mL shake flasks containing 100 mL A^+^ medium [6] in an incubator shaker (New Brunswick Innova 44) at 150 rpm and 30 °C with light:dark cycle of 16:8 h with 150 μmol m^−2^ s^−1^ light intensity, illuminated by LED lamps (Design Innova, India). For the experiment with variation of light intensity, the cultures were grown in a Multi-cultivator (MC1000-OD, PSI instruments, Czech Republic) which allows the light intensity to individual tubes to be controlled. The cultures were bubbled with air for this experiment and the temperature of the water bath was set at 38 °C, and the culture volume was 70 mL. The seeding OD for all the experiments was 0.1. Other experiments were conducted in 250 mL Dreschel (gas-washing) bottles containing 150 mL cultures. The cultures were bubbled with air, 1% CO_2_, or 3% CO_2_ in air through a gas dispersion tube with porous fritted glass tip (Sigma Aldrich Chemical Co.). The bottles were kept in a water bath maintained at 38 °C and continuously illuminated from the top with LED lights having a light intensity of 350 μmol m^−2^ s^−1^. Atmospheric air or 1% carbon dioxide in the air was bubbled into the medium at 0.5 L/min. The growth of the cells was monitored every 24 h by measuring optical density at 730 nm (OD_730_). At the end of 5 days, the culture was stopped and used to quantify various parameters.

### Plasmids construction and cloning

The genes *sbtA* (SYNPCC7002_A0470) and *bicA* (SYNPCC7002_A2371), encoding HCO_3_^−^ transporters [29], hypothetical protein genes/neutral sites NS1 (SYNPCC7002_A0935) and NS2 (SYNPCC7002_A0936) [30] and GroEL terminator were amplified from the genomic DNA of *Synechococcus* sp. PCC 7002 cells. The promoter used in this study, P_rbcL2A_, was amplified from the genomic DNA of *Synechocystis* sp. PCC 6803 while the kanR2 gene was amplified from the commercial vector pET-28 a (+), respectively. A generalized diagram of the gene cassette is shown in Fig. 7. Phusion polymerase was used for all PCR amplifications. PCR-amplified products were purified by using the GeneJET PCR purification kit. Primers used for PCR amplification are listed in Additional file 1: Tables S2 and S3. The PCR-amplified products were introduced in the commercial vector pBlueScript II SK (+) by sticky-end cloning performed using the specific restriction enzymes and T4 DNA ligase. The cloning was performed in *Escherichia coli* DH5-alpha as follows. Competent cells were prepared using a previously published protocol [31]. The transformed *E. coli* cells containing the plasmids were grown in 5 mL of LB medium in presence of ampicillin (100 µg/mL) overnight in a 30-mL test tube in an incubator shaker at 37 °C and 150 rpm. Plasmids were isolated using the GeneJET Plasmid Miniprep Kit and screened for the insertion of the desired fragment using restriction digestion.

The cassettes of interest were separated from pA and pB (the plasmids carrying the inserts for *sbtA* and *bicA*, respectively, Additional file 1: Figure S4) by double digestion. Approximately, 2 µg of plasmid DNA was digested in a 30 µl reaction mixture volume by corresponding restriction enzymes at 37 °C for 30 min to separate the cloned cassette from the backbone plasmid. Then the reaction mixture was run on 1% Agarose gel. The band of interest was excised out from the gel and the DNA was isolated using GeneJET Gel Extraction DNA Recovery kits (Thermo Scientific) in 50 µl MilliQ water (Millipore Corporation). The linear sequences thus obtained were used for the transformation of the *Synechococcus* sp. PCC 7002 cells as explained below.

### Transformation of *Synechococcus* sp. PCC 7002 cells

*Synechococcus* sp. PCC 7002 cells were transformed with the isolated DNA fragments as per earlier protocols [32] with modifications. *Synechococcus* sp. PCC 7002 was grown in A + medium until OD_730_ of ~ 1 was reached. Then, the linearized DNA fragments (~ 1 μg) were added to 800 µl culture in a microcentrifuge tube. The tube was kept in an incubator shaker (Innova 44R, New Brunswick Scientific) at 30 °C with 150 rpm and 150 μmol m^−2^ s^−1^. After 24 h, this culture was centrifuged at 2500*g* for 5 min at room temperature and the supernatant (700 µl was discarded). The resulting pellet was resuspended in 100 μL medium and spread on A^+^ agar plates containing 50 µg/mL of kanamycin monosulfate. The single colonies were re-streaked four times on the antibiotic plates to obtain complete segregants. The clones were screened for the integration of the construct in the genomic DNA by PCR. Positive transformants were selected for further analysis.

### RNA isolation and relative gene expression analysis

Gene expression analysis was done by quantitative real-time PCR (qRT-PCR). Cells were grown to exponential phase (OD_730_ ~ 1). 50 mL culture was harvested by centrifugation at 4000*g* for 5 min. The supernatant was discarded and the pellet was crushed in liquid nitrogen with an RNase-free mortar and pestle. RNA was isolated using the RNeasy Plant Mini Kit (Qiagen) as per manufacturer’s protocol. The isolated RNA was subject to DNase I treatment to remove any residual DNA contamination. 1 µg of the purified RNA was taken and cDNA was synthesized using a cDNA synthesis kit, according to the manufacturer’s protocol. The cDNA synthesized corresponding to 50 ng total RNA was used for the qRT-PCR. The qRT-PCR was performed using the KAPPA SYBR Fast Kit (Sigma-Aldrich). The gene phosphoenolpyruvate carboxylase (SYNPCC7002_A1414), *ppC* was taken as the reference gene [33], and the genes *sbtA* and *bicA* were used as the target genes for the study using the primer pairs listed (Additional file 1: Table S4). Relative expression changes were calculated as the 2^(−∆∆Ct)^ values.

### Enrichment of membrane proteins and Western blotting

Cells were grown to exponential phase, washed and resuspended in 1 mL lysis buffer [34], and lysed with 100 µL of 0.1 mm glass beads for 10 cycles of 30 s each using a FastPrep-24 beadbeater (MP Biomedicals) with alternate chilling [15, 34]. Cell debris was removed by centrifugation for 15 s at 21,000*g* at 4 °C and the supernatants were transferred to new tubes. Supernatants were centrifuged again at 21,000*g* at 4 °C for 10 min to get crude membranes in the form of pellets. Crude membrane fractions were supplemented with sodium dodecyl sulfate (SDS) sample buffer to final concentrations of 62.5 mM Tris–HCl, pH 6.8, 4% (w/v) SDS, 1 mM dithiothreitol (DTT) and 10% glycerol. Samples were incubated at 70 °C for 20 min. The protein sample was centrifuged at 21,000*g* for 15 min to remove the insolubles. The protein concentration of the resultant supernatant fraction was determined with a protein assay kit (Thermo-Scientific). The protein fractions were separated on 12% Tris–glycine protein gels by SDS-PAGE. Proteins were transferred to PVDF membrane (Bio-Rad) using the semi-dry transfer method using a Western blot apparatus (Bio-Rad) according to the manufacturer protocol. The expression of the His6-tagged SbtA and BicA was detected immunochemically with a monoclonal anti-His antibody (Sigma-Aldrich). Proteins were visualized by chemiluminescence detection with horseradish peroxidase-conjugated secondary antibody and the DAB substrate kit (Thermo-Scientific).

### Dry cell weight determination

Dry cell weight was measured as an earlier published protocol [35] with some modifications. Cell culture was centrifuged at 2500*g* for 10 min, and the pellet obtained was washed twice in 8.25 mM Tris–HCl buffer (pH 8.2). The pellet was dried at 65 °C for 36 h until the weight of the tube became constant. The dry cell weight per OD_730_ cells was calculated for various cultures.

### Measurement of glycogen content

Glycogen was measured as per previously published protocol [36], with slight modifications. 1 mL culture of cells at OD_730_ of ~ 2 was centrifuged at 4000*g* for 5 min. The supernatant was discarded, and the pellet was resuspended in 400 µl of 7.5% sulfuric acid. The mixture was heated at 100 °C for 2 h to hydrolyze the glycogen. Glucose release was measured by HPLC (Agilent Technologies) using the Aminex HPX 87H column, where 5 mM sulphuric acid was used as the mobile phase with a flow rate of 0.3 mL min^−1^.

### Extracellular (EC) carbohydrate estimation

EC carbohydrate was precipitated according to [15] with slight modifications. A 2-mL culture on the 5th day was centrifuged at 2500*g* for 10 min. The supernatant was filtered through a 0.45-µm filter (Nylon-66, MDI Membrane Technologies, India) to remove any remaining cells. Then, 2 volumes of absolute ethanol were added to the supernatant and kept at − 20 °C overnight to precipitate the carbohydrate. The tube was centrifuged at 16,000*g* for 30 min at 4 °C to pellet down the carbohydrate. The resultant carbohydrate pellet was resuspended in 200 µl of DDW and the carbohydrate concentration was determined using the phenol–sulphuric acid method according to an earlier protocol [37].

### Measurement of photosynthetic pigments

The photosynthetic pigments chlorophyll *a* and the carotenoids were measured as described in [38, 39] with slight modifications. 1 mL culture containing 2 OD_730_ cells was centrifuged in a 1.5 mL microcentrifuge tube at 4000*g* for 5 min. The pellet obtained was resuspended in 1 mL ice-cold methanol (from Sigma-Aldrich), and kept on ice in dark for 1 h to complete the extraction of photosynthetic pigments. The microcentrifuge tube was then centrifuged at 21,000*g* for 7 min at 4 °C. The supernatant was analyzed spectrophotometrically at the specific wavelengths, as suggested in the protocol used.

### Measurement of phycobiliproteins

Phycobiliproteins, viz., phycocyanin, allophycocyanin and phycoerythrin were measured with a modified protocol described in earlier studies [15, 40]. 1 mL culture of cells at OD_730_ of 2 was centrifuged and the pellet was frozen in liquid nitrogen. After thawing on ice, 1 mL ice-cold phosphate buffer saline (pH 7.2) was added and the cells were lysed with 0.1 mm glass beads using bead beater (FastPrep-24™, MP Biomedicals) for six cycles of 30 s, followed by chilling on ice (for 1 min) after each cycle. After centrifugation, supernatant absorbance was measured at 562, 615 and 652 nm using a spectrophotometer.

### Statistical analysis

All experiments were performed in biological triplicates. One-way ANOVA, followed by Bonferroni test for pairwise comparison was performed for statistical analysis (SigmaPlot version 12.5, Systat Software Inc.).

## Supplementary information

**Additional file 1: Figure S1.** *sbtA* sequence gene from *S*. 7002 (SYNPCC7002_A0470). **Figure S2.** *bicA* gene sequence from *S*. 7002 (SYNPCC7002_A2371). **Figure S3.** Ribosome binding site (RBS) sequence. **Table S1.** Genes and sources. **Table S2.** Primers Sequences for the *sbtA* cassette. **Table S3.** Primers Sequences for the *bicA* cassette. Table S4. RT-PCR Primers. **Figure S4.** Vectors maps viz. (A) pBluescript SK (+), used as cloning vector; (B) pA, vector carrying *sbtA* gene cassette; and (C) pB the vectors carrying *bicA* gene cassette.

## Data Availability

Data will be made available to all interested researchers upon request.

## Abbreviations

Ci: inorganic carbon
OD: optical density
WT: wild-type *Synechococcus* sp. PCC 7002
