# Supplementary material for: Overexpression of bicarbonate transporters in the marine cyanobacterium Synechococcus sp. PCC 7002 increases growth rate and glycogen accumulation
Source: Biotechnol Biofuels. 2020 Jan 28;13:17. doi: 10.1186/s13068-020-1656-8 (PMC6988372; doi:10.1186/s13068-020-1656-8)
Supplement: Supplementary file 1 — Additional file 1: Figure S1. sbtA sequence gene from S. 7002 (SYNPCC7002_A0470). Figure S2. bicA gene sequence from S. 7002 (SYNPCC7002_A2371). Figure S3. Ribosome binding site (RBS) sequence. Table S1. Genes and sources. Table S2. Primers Sequences for the sbtA cassette. Table S3. Primers Sequences for the bicA cassette. Table S4. RT-PCR Primers. Figure S4. Vectors maps viz. (A) pBluescript SK (+), used as cloning vector; (B) pA, vector carrying sbtA gene cassette; and (C) pB the vectors carrying bicA gene cassette. [file 13068_2020_1656_MOESM1_ESM.docx]

**Additional data for article**

Overexpression of carbon transporters in cyanobacteria increases growth rate and glycogen accumulation

**Figure S1.** *sbtA* sequence gene from *S*. 7002 (SYNPCC7002_A0470).

GTGGATTTTTTGTCCGATTTTTTGACGAAATTTGGGTCGCAGTTGCAGTCCCCGACGCTC

GGCTTTTTAATTGGCGGTATTGTCATTGCCGCCTTCGGTAGCCGACTTACAATCCCCGAT

GCAGTGTATAAGTTCATCGTTTTTATGCTGCTCATCAAAGTCGGTTTGAGCGGCGGTATT

GCGATTCGTAATACCAATATCACGGAGATGCTCTTGCCTGCGTTATTTGCTGTGCTCATG

GGCATTCTGATCGTTTTTATTGGGCGTTTTACCTTAGCAAAGCTGCCAGGTATTAGGACC

GTAGATGCAGTGGCAACTGCCGGCTTGTTTGGGGCAGTGAGTGGTTCGACCCTTGCTGCT

GGAATCACGGTTATGGAAGGGCAAGGTGTTTTCTACGAACCTTGGGCAGCGGCACTTTAT

CCTTTTATGGATATTCCCGCCCTGGTGACAGCGATTGTTGTAGCTAGTCTTTATAAGAGC

AAGCAGCGCGAGGTTGAAGCCGATGATTTCAGCAAACAACCCGTTGCCGCTGGTGAATAT

TCTGGTGAACCTGTTTATCCCACCACGAGGCAGGAATATCTGGGTCAAAAGCGTGGTAAG

GCTACTAATCGGGTTGAAATTTGGCCCATTGTTAAGGAAAGTCTCCAGGGTTCTGCCCTA

TCAGCATTGTTGCTCGGTCTTGCTCTCGGTTTGTTGACTCGGCCAGAAAGTGTCTTTGAA

AGTTTCTATGAGCCCCTCTTCCGTGGTTTTCTTTCGATTTTGATGCTGGTGATGGGGATG

GAAGCTTGGTCTAGGCTTGGGGAACTGCGCAAAGTTGCTCAATGGTACGCTGTCTATGCG

TTTATTGCGCCGCTACTCCATGGGTTTATTGCCTTCGGTCTCGGCATGATCGCCCACTAT

GTTACAGGGTTCAGTCCTGGTGGTGTTGCCCTCTTAGCGATTATTGCGGCGTCTAGTTCG

GACATCTCTGGGCCGCCTACTTTACGGGCTGGGATTCCGTCGGCTAATCCTTCTGCTTAT

ATCGGTTCGTCTACGGCCATCGGTACCCCCGTGGCGATCGCCATCGGCATACCACTTTTT

ATCGGCCTTGCCCAAGCAACCATGGGTGGCTGA

**Figure S2.** *bicA* gene sequence from *S*. 7002 (SYNPCC7002_A2371).

ATGCAGATAACCAACAAAATTCACTTTAGGAATATCCGCGGCGATATTTTTGGCGGGCTA

ACGGCGGCGGTCATTGCGTTGCCCATGGCCCTCGCCTTCGGGGTGGCATCCGGTGCCGGG

GCAGAAGCCGGTCTCTGGGGTGCTGTGCTTGTGGGCTTCTTTGCCGCCCTCTTTGGGGGA

ACCCCCACCCTCATTTCCGAACCCACAGGGCCGATGACGGTGGTTATGACCGCTGTGATT

GCCCATTTCACGGCCAGCGCCGCTACTCCAGAAGAAGGTTTGGCGATCGCCTTTACCGTC

GTGATGATGGCCGGGGTGTTCCAAATTATTTTTGGCTCCCTCAAACTCGGCAAATACGTC

ACCATGATGCCCTACACCGTGATTTCTGGCTTCATGTCAGGGATCGGGATCATCCTGGTC

ATTTTGCAATTAGCGCCCTTCCTGGGACAGGCGAGTCCGGGGGGCGGCGTCATCGGCACG

CTCCAAAATTTACCCACACTGCTGAGTAATATTCAACCGGGCGAAACAGCCTTAGCTTTA

GGCACCGTGGCGATCATCTGGTTTATGCCAGAGAAGTTTAAAAAGGTAATCCCGCCCCAA

TTGGTCGCCCTTGTCTTGGGGACAGTAATCGCCTTTTTTGTTTTCCCGCCAGAAGTAAGC

GATCTCCGTCGCATCGGTGAAATTCGAGCTGGGTTCCCAGAGCTGGTCAGACCGAGCTTT

AGTCCGGTTGAATTCCAGAGAATGATCCTCGATGCGGCAGTGCTAGGGATGCTCGGTTGT

ATCGATGCCCTCTTGACGTCTGTCGTCGCCGATAGCTTGACCCGGACAGAGCATAATTCC

AACAAAGAATTAATTGGCCAAGGTCTAGGGAACCTCTTTTCTGGCTTGTTCGGCGGGATT

GCTGGGGCTGGGGCCACCATGGGGACTGTGGTAAATATCCAGTCCGGTGGTCGAACGGCG

CTTTCTGGATTGGTACGGGCCTTTGTCCTGTTGGTTGTGATCCTTGGGGCGGCTAGTTTA

ACGGCAACCATCCCCCTGGCTGTGCTTGCTGGGATTGCCTTCAAGGTCGGGGTTGACATC

ATTGACTGGAGTTTCCTAAAACGGGCCCACGAAATTTCCCCCAAGGGGGCACTGATCATG

TACGGCGTCATTCTGTTGACGGTCTTAGTTGACTTGATTGTCGCCGTGGGTGTGGGTGTG

TTTGTCGCTAATGTGCTCACCATCGAACGGATGAGTAATCTCCAGTCTGAAAAAGTCCAA

ACGGTTAGTGATGCTGACGATAATATCCGCCTGACTACCACTGAAAAACGCTGGTTGGAT

GAAGGCCAAGGCCGTGTTCTTTTGTTTCAACTCAGTGGGCCGATGATCTTTGGGGTCGCA

AAGGCGATCGCCAGAGAACACAATGCGATGGGTGACTGTGATGCCCTCGTCTTTGATATC

GGTGAAGTGCCCCACATGGGGGTTACCGCTTCCCTAGCCTTAGAAAATGCCATTGAAGAG

GCCCTCGACAAAGAACGTCAGGTTTATATTGTCGGTGCTGCGGGCCAAACCCGTCGCCGT

CTGGAAAAACTCAAGCTCTTTAAGCGGGTTCCCCCCGATAAATGTTTGATGTCGCGGGAA

GAAGCCCTCAAGAATGCCGTGCTCGGAATCTATCCCCATTTGGCGGATGGTGTTACGGCT

CCCAGTTCAGAGATGGGTTAA

**Figure S3. Ribosome binding site (RBS) sequence**

AGGAGA

**Table S1. Genes and sources**

| **Gene** | **Source** |
| --- | --- |
| NSI/SYNPCC7002_A0935 | S.7002 genomic DNA |
| PrbcL2A | S.6803 genomic DNA |
| sbtA | S.7002 genomic DNA |
| bicA | S.7002 genomic DNA |
| kanR2 | Vector pET-28a(+) |
| GroEL Ter | S.7002 genomic DNA |
| NSII/ SYNPCC7002_A0936 | S.7002 genomic DNA |

**Table S2. Primers Sequences for the *sbtA* cassette**

| **Primer Name** | **Sequence** |
| --- | --- |
| **NSI-FP** | CAGTACTCGAGATGTTTGGTCGAAAATTCTTCACACT |
| **NSI-RP** | CAGTAGTCGACTTACTCAGTTTTTAAGTAATTAGCAGA |
| **P-FP** | CAGTAGTCGACTCACCATTTGGACAAAACATCAGC |
| **P-RP** | CAGTAGATATCTTAAACATTGAATAGCCTAGCTTTCT |
| **sbtA-FP** | CAGTAGATATCAGGAGAAAACCGCGTGGATTTTTTGTCCGATT |
| **sbtA-RP** | CAGTAGAATTCTCAGTGGTGGTGGTGGTGGTGGCCACCCATGGTTGCT |
| **kan-FP** | CAGTAGAATTCGGAGGAAAACCGCATGCACCACCACCACCACCACATGAGCCATATTCAACGGGAAAC |
| **kan-RP** | CAGTAGGATCCTTAGAAAAACTCATCGAGCATCAAA |
| **Ter-FP** | CAGTAGGATCCGGTTTAGTGACCGACTAACACTTT |
| **Ter-RP** | CAGTAACTAGTAAACGAAAGAGAGCTTAGCAAGTG |
| **NSII-FP** | CAGTAACTAGTCTATTTTATCTCGTTTTCTTCCCAG |
| **NSII-RP** | CAGTAGAGCTCATGAATAAACTGTTTACAGCAGCC |

**Table S3. Primers Sequences for the *bicA* cassette**

| **Primer Name** | **Sequence** |
| --- | --- |
| **NSI-FP** | CAGTAGGTACCATGTTTGGTCGAAAATTCTTCACACT |
| **NSI-RP** | CAGTACTCGAGTTACTCAGTTTTTAAGTAATTAGCAGA |
| **P-FP** | CAGTACTCGAGTCACCATTTGGACAAAACATCAGC |
| **P-RP** | CAGTAGTCGACTTAAACATTGAATAGCCTAGCTTTCT |
| **bicA-FP** | CAGTAGTCGACAGGAGAAAACCGCATGCAGATAACCAACAAA |
| **bicA-RP** | CAGTAAAGCTTTTAGTGGTGGTGGTGGTGGTGACCCATCTCTGAACTGGGAG |
| **kan-FP** | CAGTAAAGCTTGGAGGAAAACCGCATGCACCACCACCACCACCACATGAGCCATATTCAACGGGAAAC |
| **kan-RP** | CAGTAGGATCCTTAGAAAAACTCATCGAGCATCAAA |
| **Ter-FP** | CAGTAGGATCCGGTTTAGTGACCGACTAACACTTT |
| **Ter-RP** | CAGTATCTAGAAAACGAAAGAGAGCTTAGCAAGTG |
| **NSII-FP** | CAGTATCTAGACTATTTTATCTCGTTTTCTTCCCAG |
| **NSII-RP** | CAGTAGAGCTCATGAATAAACTGTTTACAGCAGCC |

**Table S4. RT-PCR Primers**

| **Primer Name** | **Sequence** |
| --- | --- |
| **rtppC-FP** | CATCGGCCGCTTCTACTTTAT |
| **rtppC-FP** | ATCGTTACCGCCAAGAACCC |
| **rtsbtA-FP** | CGATTTTGATGCTGGTGATG |
| **rtsbtA-RP** | GCCGCAATAATCGCTAAGAG |
| **rtbicA-FP** | GTCCGGTTGAATTCCAGAGA |
| **rtbicA-RP** | CGAACAAGCCAGAAAAGAGG |

**A**

**B**

**C**

**Figure S4:** Vectors maps viz. (A) pBluescript SK (+), used as cloning vector; (B) pA, vector carrying *sbtA* gene cassette; and(C) pB the vectors carrying *bicA* gene cassette.
